# The impact of stigma on people with albinism in Africa: a narrative review

**DOI:** 10.1007/s12687-026-00872-0

**Published:** 2026-03-21

**Authors:** Jennifer GR Kromberg, Robyn A Kerr

**Affiliations:** Division of Human Genetics, School of Pathology, Faculty of Health Sciences, University of the Witwatersrand and National Health Laboratory Service, Johannesburg, South Africa

**Keywords:** Africa, Oculocutaneous albinism, Psychosocial, Stigma, Stigmatisation, Discrimination

## Abstract

Oculocutaneous albinism (OCA) is a recessively inherited condition which affects about 1 in 5,000 people in Africa. The depigmentation of the skin and hair is very striking, unusual and unexpected, and stigmatisation has been ongoing for centuries. This narrative review asked: How does stigma related to OCA manifest in African settings, what drives it, what impacts does it have across health, education, psychosocial well-being and human rights, and what interventions have been proposed or implemented to reduce harm? The review was conducted using searches of PubMed and Google Scholar (up to June 2024), supplemented by targeted retrieval of policy and NGO reports; 60 sources met inclusion criteria and were synthesised thematically. Results showed that widely held cultural beliefs and myths, that originate in perceptions of difference cause, and perpetuate, stigmatisation. Maternal-infant bonding, schooling, social interactions, health, marriage, employment issues, personal safety and human rights may all be affected by the stigmatising behaviour of the community. Children as well as adults may be attacked for their body parts, believed to make powerful medicine in some countries. Healthcare workers’ stigmatising attitudes may result in poor delivery of essential health care services. Services that should be provided for people with albinism include appropriate healthcare, genetic counselling, and possibly general counselling to provide support and guidance for managing the condition and coping with the issues associated with endemic stigmatisation. In conclusion, stigma related to OCA in Africa is driven primarily by socio-cultural beliefs and reinforced by structural inequities, producing wide-ranging harms across the life course. Awareness programs are required to inform the public and dispel myths, specialised healthcare services need to be increased, and guidelines on managing albinism should be provided to health and educational professionals, so that incidents of stigmatisation may be minimised.

## Introduction

Albinism is a group of inherited pigment conditions in which melanin synthesis is reduced or absent (Summers 2009). Among these, oculocutaneous albinism (OCA) is one of the most common autosomal recessive genetic conditions in Africa, occurring in approximately 1 in 5,000 sub-Saharan Africans on average, compared to an estimated 1 in 10,000 to 15,000 Europeans, depending on the population group (Kromberg et al. 2023). Phenotypically, people with OCA have both skin and eye defects, resulting in a predisposition to skin cancer and poor visual acuity. In Africa, the unusual and striking white skin and hair colour is obvious at birth (Kromberg et al. 1987). By contrast, in many European populations, the condition is more often diagnosed later in childhood, when visual impairments become evident (Froggatt 1960; Healey et al. 2014).

There are many unique psychosocial issues that surround individuals with genetic disorders. When the disorder is obvious to the onlooker and perceived as very different from the normal, the affected person can become stigmatised. The source of this stigmatisation is not entirely associated with the views and constructs in the onlooker, but also with his or her background, local community attitudes and cultural experiences. Attitudes and reactions toward physical deviation, as well as the apprehension and reactions people have to the different and unusual, can influence the development of stigmatisation (Wright 1960).

To have a genetic disorder and to be different is to be set apart, a basis for labelling, stereotyping and exclusion. Such processes are described in quantitative work, from Nigeria and elsewhere, as central to lived experiences of stigma (Olagunju et al. 2025). Conformity is valued in human society and people tend to feel greater affinity toward those they perceive as similar to themselves (Heider 1958). Goffman (1963) suggested that stigmatisation was a social process, not a personal trait, and it arose when society labelled some characteristics as undesirable and devalued some individuals based on perceived differences. Building on this theory, Link and Phelan (2001) conceptualised stigma as a systematic social process comprising labelling, stereotyping, separation, status loss and discrimination, all enacted within contexts of social, economic and political power. More recent frameworks have refined classic theories by distinguishing types and levels of stigma which include: public (enacted) stigma, referring to discriminatory acts and attitudes (often based on cultural beliefs); structural stigma, encompassing systemic and policy-level discrimination; internalised stigma, occurring when individuals accept and internalise negative stereotypes; perceived or felt stigma, involving fears of rejection (even in the absence of overt discrimination); and stigma by association, negative attitudes directed towards family members (Hatzenbuehler and Link 2014; Stangl et al. 2019). Stigma can manifest at cultural and institutional levels as well as interpersonal and individual levels, each reinforcing the other. Stigmatisation is a changing social process which can only be understood as it occurs during social interactions and in certain contexts (Parker and Aggleton 2003). The process can have a huge (and probably underestimated) impact on the stigmatised person’s life, affecting opportunities such as schooling, employment, relationships, housing and access to medical care (Link and Phelan 2001). The chronic stress often associated with stigma can have negative effects on both health and general well-being (Link and Phelan 2006). For people with albinism in Africa, these frameworks show that stigma stems from cultural interpretations of difference, perpetuated by institutional neglect, and internalised through ongoing social marginalisation and invisibility.

People with OCA have been described and investigated over the centuries by explorers, missionaries, anthropologists and medical practitioners, in many different African countries. The comprehensive monograph published by Pearson et al. (1913) covers worldwide reports collected prior to 1913. One of the earliest, written in the first century AD, is that of Plinius Secundus (1942 translation), who described white Ethiopians living in North Africa. They were very pale, had black parents, disliked the sun and avoided people, as if their albinism was contagious. In Guinea, people with OCA were considered a great misfortune for the family, and in Gabon, affected individuals were seen as unlucky and frequently killed, unless missionaries intervened. Historical accounts describe infanticide of infants born with albinism in certain African countries, alongside banishment and social exclusion (Pearson et al. 1913). More recent reports, such as that from Ghana, describe continued instances of infanticide, brutalisation of individuals with OCA, and ostracisation from communities (Daklo and Obadire 2024). Discrimination varies between various African countries and is not always negative. Reports from the Sudan suggested that people with albinism were treated preferentially and not expected to work (Raffenel, quoted by Pearson et al. 1913). In the Congo, people with albinism have been described as sacred and inviolate, ‘spirit children’ (Steyn 2022). The current reality, however, indicates that people with the condition are still being stigmatised in many different African countries (Braathen and Ingstadt 2006; Ojilere and Saleh 2019; Olagunju et al. 2025; United Nations Report to the General Assembly 2019).

The psychosocial burden of stigmatisation is particularly heavy in Africa, and the stigmatising is often overt and can cause low self-esteem, identity conflicts, and internalised shame in the affected individual and family (Bradbury-Jones et al. 2018; Kromberg and Jenkins 1984). In contrast, the impact in high-income countries may be more covert and associated mainly with low vision in the individual (Fournier et al. 2024). Due to the impact of stigmatisation of people with albinism living in Africa, as observed by the authors, and the limited published articles on the topic, the present investigation was conceived. This narrative review asks: How does stigma related to OCA manifest in African settings, what cultural and structural mechanisms drive it, what are its psychosocial, educational, health, and human-rights impacts across the life course, and what interventions have been proposed or implemented to mitigate these harms? During preliminary scoping of the literature, the question was refined from a broad focus on ‘stigma and albinism’ to a more specific focus on OCA in Africa, because the majority of African literature addresses OCA, and stigma is frequently linked to visible difference and associated beliefs in African contexts.

### Methodology

This review was conducted as a narrative review, synthesising selected published evidence on the different dimensions and consequences of stigma experienced by people with albinism in Africa. A narrative approach was chosen, given the diverse nature of available evidence (ranging from qualitative studies, ethnographic and historical accounts, biomedical and public health research, policy documents, and NGO/UN reports) that are not readily amenable to a single systematic design or meta-analysis.

**Databases and rationale**. Electronic searches were performed in PubMed and Google Scholar for publications available up to June 2024. PubMed was selected to capture biomedical and public health research (including dermatology, ophthalmology, genetics, and health services research). Google Scholar was selected to broaden coverage to social science literature and to identify relevant grey literature (e.g., NGO and UN reports) that may not be indexed in PubMed.

**Search strategy.** The following search terms were used: ‘albinism AND stigma’, ‘albinism AND discrimination’, ‘albinism AND Africa’, ‘psychosocial AND albinism’, ‘beliefs OR myths AND albinism’. The article title was also used as a prompt in an AI-assisted search tool to identify potentially relevant literature, which was then verified and sourced from original peer-reviewed publications and reports. The relevant articles were hand-searched to identify additional sources, including anthropological and historical accounts. Also, the author’s many years of experience in the field provided further meaningful references and information (Kromberg and Kerr 2025).

**Eligibility criteria.** Publications were included if they: referred to OCA/albinism in African contexts and described any form of stigma (public/enacted, internalised, perceived/felt, structural/institutional, or stigma by association), its drivers, consequences, or interventions. Eligible publication types included peer-reviewed research, case studies, ethnographic and historical accounts, policy documents, and high-credibility reports. Publications were excluded if they: did not address stigma-related phenomena or focused on non-African contexts without clear relevance for the review question.

**Selection and synthesis.** Titles and abstracts were screened for relevance to the review question, followed by full-text review of eligible sources. Key information was extracted (location, design, population, stigma mechanisms and manifestations, and reported impacts/interventions). A thematic synthesis approach was used to organise findings into six recurrent themes: (i) Cultural roots of stigma; (ii) The psychosocial impact of stigma; (iii) Manifestations of stigma: health, education and employment issues; (iv) Violence and human rights; (v) Coping and resilience; (vi) Interventions and advocacy.

**Included sources.** In total, 60 sources were included and synthesised across six thematic areas.

## Findings and discussion

(i) Cultural roots of stigma

The stigmatisation of albinism in Africa originates from ancient interpretations and community perceptions of difference (Imafidon 2019). This difference is not only seen as physical, but also as ontological and associated with basic ideas about the nature of being. Historically, many ethnic groups associated the cause of albinism with their spiritual frameworks, sometimes as divine punishments for transgressions or omens of misfortune, but occasionally as a spiritual blessing (Kromberg and Jenkins 1997; Baker et al. 2010).

Various factors, specific to African cultures, perpetuate these beliefs and underpin stigmatising behaviour. Firstly, the myth that people with OCA have supernatural powers is still widely held. Many believe that they do not die natural deaths but disappear at the end of life (Kromberg 1992). Secondly, medicines made from the body parts of people with OCA are believed to have spiritual powers and to bring health, good luck and power to the consumer (Bryceson et al. 2010). This myth has led to attacks and murders in sub-Saharan African countries, particularly Tanzania. Thirdly, the genetic cause of albinism is widely misunderstood (Braathen and Ingstad 2006). In a South African study, 69% (43/62) of participants (both people with albinism and controls) did not know what caused albinism, another 18% (11/62) gave the cause as divine intervention, and only 5% (3/62) mentioned genetic causes (Kromberg and Jenkins 1984). A more recent study, in Uganda, presented similar findings and reported causes included the mother being impregnated by a ghost, or having laughed at a person with albinism, or being punished by God for bad deeds; only a few participants accurately identified albinism as an inherited genetic condition (Bradbury-Jones et al. 2018). Cruz-Inigo et al. (2011) suggested that socioeconomic factors, such as poverty and illiteracy, may result in the lack of awareness of albinism and its aetiology in Africa and contribute to the development of stigma. These studies showed a low awareness of the genetic causation, and there is a lack of research to indicate whether there has been improvement in knowledge. More positive attitudes towards people with the condition have resulted from health education advocacy and NGO involvement; however, literacy still varies by region, education and socio-economic status (Franklin et al. 2018; Daklo and Obadire 2024).

(ii) The psychosocial impact of stigma

Published articles on albinism and stigmatisation, specifically in Africa, are sparse. However, some psychosocial aspects of albinism, such as family attitudes, adjustment, self-concept and identity, intelligence, quality of life, cultural beliefs, and education have been studied (e.g. Kromberg 2018a, b; Braathen and Ingstad 2006). The development of psychosocial issues was observed initially in a study on mothers with newborn infants with albinism (Kromberg et al. 1987). The mothers had difficulty relating to the infant (implying some internalised and associated stigma), who was so phenotypically different from themselves, and maternal-infant bonding was significantly delayed. However, by three months, such behaviour had lessened. These results show that although shock, cultural background and personal beliefs may affect the initial reactions of most mothers, giving rise to stigmatisation, such behaviour may be modified over time.

In 1989, Ezeilo studied psychosocial aspects of albinism in Nigeria. His three student participants commented that their skin colour attracted attention, resulting in stigmatisation, name-calling, and ridicule (suggesting both public and internalised stigmatisation). This experience led to their withdrawal from social situations (to avoid being noticed); they regarded society as unkind and rejecting. The participants in a recent Malawian study confirmed that physical appearance is often integral in how interaction happens in social groups (Tambala-Kaliata et al. 2021), and how people with albinism become stigmatised. The consequences of stigmatisation in Africa are far-reaching and dealing with stigma is a lifelong negative experience for most people with albinism (Likumbo et al. 2021).

(iii) Manifestations of stigma: Health, education and employment issues

People with OCA face systemic barriers in many different areas of their lives. Such barriers often result in health-related stigma, associated with poor access to healthcare services, and structural stigma related to the social determinants of health (Reimer-Kirkham et al. 2022). This Tanzanian study reported on the stigmatising attitudes and behaviour of healthcare professionals, the lack of access to essential skin and eye care, the absence of any relevant health education, and gender inequality associated with increased stigmatisation. In Zimbabwe, healthcare was also found to be poor and less than half the children (45.7%, 63/138) with albinism (mean age 14.4 years) studied had had eye testing, and only 24.6% (34/138) had prescribed spectacles, while only 23% (32/138) had ever had their skin examined (Lund 2001).

Systemic barriers may also cause educational difficulties. The visual impairment is problematic in the classroom, and without attention to their needs, most affected students cannot read at standard distances (Clarke and Beale 2018). Bullying may occur, and South African students with albinism stated that some of their peers bullied, rejected, and/or avoided touching them, fearing contagion (Phatoli et al. 2015). In a Tanzanian study, students with albinism (68%, 101/149) found that their low vision affected their education severely, and 56% (89/149) only had primary education, while 8% (12/149) had no formal education at all (Kiprono et al. 2012). Further, early dropout occurred, and students with albinism had a 63% lower secondary school completion rate than their peers in another Tanzanian study (Franklin and Lund 2017). This trend is partly due to the difficulty in managing schoolwork with a visual impairment, partly to the stigmatising attitudes of their peers and partly to the inability to participate in outdoor activities due to sun sensitivity (Franklin et al. 2018; Kromberg et al. 2020). However, children with albinism in Nigeria coped with problems at school by developing ‘amplified listening skills’ to help them manage in the classroom, and by joining the local albinism group for support (Olagunju et al. 2025).

Economic marginalisation, probably caused by public and structural stigmatisation, may also occur, and people with albinism are at a disadvantage due to their low vision and sensitive skin, which prevents them from doing farm work in rural areas (Bryceson et al. 2010) or any outdoor work in general. Also, employers may fear bad luck and contagion if they employ them (Baker et al. 2010), and there are limited suitable vocational training opportunities available in most African countries. The findings of a Botswana study showed that 54% (27/50) of the affected individuals studied were unemployed, and 20% (10/50) relied on government welfare support (compared with 2% (2/99) of people without albinism in that country), while the earnings of those with albinism were significantly less than those without the condition (Chu et al. 2021).

(iv) Violence and human rights

Manifestations of stigma are evident in the recent increase in human rights abuses and physical attacks. Under The Same Sun (2024) reported that 731 attacks, including 204 killings, had been documented across 31 African countries. This document emphasises that many attacks are not reported, and these figures are likely a gross underestimation. Most victims are children (Taylor et al. 2019). Grave robberies have also occurredmostly for the illegal acquisition of body parts (Franklin and Lund 2017).

Research shows that African women and girls with OCA are disproportionately stigmatised (Reimer-Kirkham et al. 2022; Olagunju et al. 2025; Ojilere and Saleh 2019). The mother may be blamed for delivering a ‘white’ baby, accused of infidelity, rejected by the father’s family and sometimes abandoned (Kromberg et al. 1987; Baker et al. 2010). Such gender-based inequality often leaves mothers with a lack of family support and few resources (Heymann et al. 2019). Further, women with OCA may be targeted as sexual partners, as there is a myth that intercourse with such women can cure HIV/AIDS (Ojilere and Saleh 2019). Also, affected girls were found to have been denied schooling more often than boys in a Zimbabwe study (Lund 2001). However, both men and women with albinism are often rejected as marriage partners, and 52% (mean age 32 years) were still single in one Tanzanian study (Kiprono et al. 2012), while 10/11 adult participants were single in a recent Nigerian study (Olagunju et al. 2025).

(v) Coping and resilience

Sociological research on how people with OCA cope with stigmatisation is limited. It is assumed that many affected individuals worldwide experience stigmatisation. The most comprehensive report on albinism and coping that is available was published by Wan (2003) on 12 participants living in Canada, the USA and Australia. Like the experiences reported from Africa (Malawi, Likumbo et al. 2021; Tambala-Kaliata et al. 2021), the respondents described name-calling, humiliation and victimisation from students and teachers in primary school, resulting in unhappiness and social isolation (public stigmatising); less discrimination occurred at high school, where students and staff gained understanding. Nevertheless, dating was difficult, there was marginalisation in the workplace (structural stigmatisation), and respondents also found many health professionals to be insensitive and ignorant. To manage these adversities, Wan (2003) observed that people with OCA developed coping mechanisms. These included: being defiant, active, flamboyant and/or positive (accepting albinism as part of their identity); cultivating serenity; internalising; talking; and hiding. Wan (2003) concluded that a variety of ways of dealing with stigmatisation are necessary for the personal growth and well-being of people with OCA.

Ezeilo (1989), working in Nigeria, commented that coping with albinism depends on both the family environment and socialisation processes. If children are nurtured as individuals, not as stereotypes, raised to be self-confident and assertive, then they can strive for self-actualisation and an independent self, instead of succumbing to the negative attitudes and stigmatising behaviour of society. Stigma can also be successfully coped with through forming specific identities and opinions associated with the social factors originating in the person’s family, community and informal support groups (Brocco 2016).

The lived experience of Nomsa Ndlovu (pseudonym) is illustrative of an African person with OCA who coped with lifelong stigmatisation (Mazibuko and Kromberg 2018). She was born the fourth child with OCA to caring and supportive parents. In documenting her personal journey, it is noted that it was only in her teenage years that she understood the genetic cause of albinism and started accepting herself as a ‘normal’ person, and her internalised stigmatisation (and cultural stigma) was reduced. The most intense experiences of stigma occurred during her higher education, when she was away from home, and early in her teaching career, when she was denied promotion, reflecting the structural stigma embedded in these environments. These transitional life stages were also described as particularly challenging by participants in Bradbury-Jones et al. (2018). However, she demonstrated resilience, managed her circumstances effectively, and went on to become a leader in the advocacy field. One of her daughters explained that she always tells her friends about her mother’s albinism before they meet her, to prevent the discomfort and typical discriminatory behaviour that mother and daughter have both faced on numerous occasions, a form of stigma by association.

(vi) Interventions and advocacy

Interventions aimed at changing stigma need to focus on two approaches, which must be multifaceted and multilevel (Link and Phelan 2001). Firstly, the many mechanisms giving rise to discrimination should be addressed at the individual and structural levels. Secondly, change must address the cause of the stigma, which, in Africa, means changing the deeply embedded attitudes and cultural beliefs of power groups, that result in the labelling, stereotyping, isolating, devaluing and discriminating behaviour that occurs.

Various governmental actions are contributing to reducing stigma. For example, Tanzania has taken measures against witchcraft practices. Traditional healers have been registered, and those suspected of using body parts of people with OCA have their licence to practice suspended (Brocco 2016). These actions, together with police interventions, have significantly reduced murders of people with OCA. Malawi developed a National Response Plan in 2016, and this includes legal counsel for investigations into crimes against affected people and public condemnation of such acts by top officials (Tambala-Kaliati et al. 2021). The African Union Plan of Action (2021–2023) was a continent-wide policy addressing protection, accountability, equality for persons with albinism and the eradication of discrimination and violence (African Albinism Network). Further, the appointment of the United Nations Independent Expert on Albinism in 2015 has led to groundbreaking advocacy and action plans to address stigmatisation globally (Clarke and Beale 2018; Miti-Drummond, personal communication 2025).

Other efforts include the initiation, by the United Nations in 2014, of an International Albinism Awareness Day, which raises global awareness (Brocco 2015). Also, influential NGOs, such as Standing Voice (Clarke and Beale 2018) and Under the Same Sun (UTSS) (Brocco 2015), initiate and promote appropriate health interventions that improve the lives of vulnerable persons, distribute educational materials and hold public education campaigns, which contribute to changing local attitudes.

Education reforms are being introduced, and, for example, teachers have been trained in Tanzania to deal with the low vision problems of children with OCA in schools (Kammer and Grant 2014), and guidelines for optimising vision for children in the classroom have been published (Kammer 2018). Access to eye care is also being improved, and in 2016, the Tanzanian government launched a “Vision for Life” campaign to meet the needs of 4200 children with albinism in 6 regions of the country (Franklin and Lund 2017).

Recent research showed that mothers with a newborn infant with OCA were often ignored and rejected by health professionals in Tanzanian maternity wards (Reimer-Kirkham et al. 2022). However, intervention was successful: after the staff were informed by talks on albinism from affected people, they could explain the condition to the mother, contribute to the process of destigmatisation, and provide more ethical care to their patients. Similarly, contact interventions, provided by spokespersons with albinism, offer community groups educational talks, which raise awareness, provide information, reduce stigmatisation, demystify worldviews and increase empathy (de Groot et al. 2019).

Despite these and other interventions, there are still persistent challenges and unresolved issues, including healthcare gaps which are still apparent (Franklin and Lund 2017; Tambala-Kaliati et al. 2021); genetic counselling, which is generally unavailable (Kromberg 2018a, b); and some dependency on NGOs, with many community programs relying on foreign funding, risking their sustainability.

### Limitations

The findings from the present study are limited by the few relevant documented reports that have specifically covered stigmatisation and albinism in African communities. Consequently, some old studies, and others in which stigma was first mentioned, have been included (e.g. Pearson et al. 1913; Kromberg et al. 1987). Although the study covers some early reports, the continuity of stigmatisation throughout the centuries, and throughout the continent, is apparent in such diverse articles as, for example, that by Pliny in the first century AD, on albinism in Ethiopia (describing the avoidance of people by those with albinism), and that by Daklo and Obadire in 2024, on the situation in Ghana (describing the high level of socio-cultural exclusion). It is noteworthy that many of the reports included in Pearson et al.’s monograph (1913) are from African countries from which no further relevant reports have been published (e.g. Guinea, Gabon, Sudan), as far as can be ascertained.

The study may also be biased, since it relied upon a narrative rather than a systematic methodology. Further, the authors have only consulted English-language reports, and the focus has mostly been on Southern and Central African countries (where the authors have had experience), and some of the information from other African countries might have been missed. It is acknowledged that the situation regarding persons with albinism is not the same in every African country, and there are inevitably individual differences, which cannot be teased out in a short article such as this one.

## Recommendations

From this review of the relevant literature, various recommendations can be made regarding the reduction of stigmatisation in Africa. On the personal/individual level, in countries where abuse and attacks are occurring, people with OCA require legal protection, and the attackers require identification, prosecution and punishment. Women and children are particularly susceptible to attacks, and gender issues need attention (Ojilere and Saleh 2019; Reimer-Kirkham et al. 2024). At the same time, efforts need to be made to provide better education opportunities for children (by upskilling teachers (see Kammer 2018) and employment opportunities, with suitable vocational training, for adults. Access to appropriate healthcare services, such as anti-actinic skin cream (attempts to get skin cream added to the WHO list of essential medicines have now been successful, and it now needs to be made available across the continent (Miti-Drummond, personal communication 2025) and eye-care (see Kammer and Grant’s 2015 program) need to be improved. Also, appropriate Guidelines for Managing Albinism need to be circulated widely (e.g. Moreno-Artero et al. 2021; Kromberg et al. 2020).

People with OCA, as well as their parents, should be offered genetic counselling, so that they understand the cause and management of albinism and the chance of passing it on to the next generation (Kromberg 2018a, b; Reimer-Kirkham et al. 2024). They can then explain albinism to others, in this way helping to dispel the myths around causation (Taylor et al. 2021). Genetic counselling has been shown to be beneficial and empowering and should be provided across Africa (Morris et al. 2015). Personal counselling is also recommended, since it can be psychologically empowering. Recipients can discuss their personal issues, learn to recognise local negative attitudes and stigmatisation, and how to cope with them, and may then become effective albinism advocates.

On the public and community level, awareness programs are essential, particularly those that target community leaders and influencers. Health professionals also need input, during their training, on how to manage the healthcare needs of people with albinism and prevent the serious stigmatising side-effects of untreated health issues. Lastly, local lay albinism organisations should be encouraged and supported as they provide much of the initial help and information required by affected people in the community.

Thomas (2024) suggests that although research on stigma has a long history, the concept as it is used in health and illness today needs more clarification. The term is often used in academia, but ordinary people might describe their experiences as being discriminated against, isolated, stereotyped, shamed or judged. In certain circumstances, stigmatisation is assumed, while the individual differences and personal factors underlying it in any specific setting are unrecognised. Further research into these factors is recommended, so that programs aimed at reducing stigma, on the public as well as on the private level, can become better informed and more relevant. Future research programs on albinism could be enhanced by the application of Stangl et al.’s (2019) Health and Discrimination Framework; once the drivers and facilitators of stigmatisation are better understood, more effective responses can be developed towards managing stigma in this field.

If these recommendations could be followed up on and appropriate programs put in place throughout Africa, people with albinism and their families would benefit exponentially, and their stigmatisation within the community would be reduced.

## Conclusion

There is a complex interaction between albinism and stigmatisation, and the personal characteristics and abilities of affected individuals, their families, and their environment should be taken into consideration in rehabilitation programs in Africa. It is also essential to understand the influence of deeply rooted cultural beliefs and the underlying causes of stigma in each context. Myths must be challenged, and the acceptance of difference encouraged through widespread awareness initiatives. In addition, essential health services such as skin and eye care, as well as genetic counselling, need to be more widely available and accessible. Although some progress has been achieved through the efforts of advocacy groups, albinism associations, government and NGO programmes, these initiatives require further expansion to meaningfully improve the quality of life for people with albinism. 

## Data Availability

No datasets were generated or analysed during the current study.
